# Should activated charcoal be given after tramadol overdose?

**DOI:** 10.1186/2008-2231-21-46

**Published:** 2013-06-06

**Authors:** Hamid Khosrojerdi, Reza Afshari, Omid Mehrpour

**Affiliations:** 1 Addiction Research Center, Imam Reza Hospital, School of Medicine, Mashhad University of Medical Sciences, Mashhad, Iran; 2 Birjand Atherosclerosis and Coronary Artery Research Center, Birjand University of Medical Science, Birjand, Iran; 3 Medical Toxicology and Drug Abuse Research Center (MTDRC), Birjand University of Medical Sciences (BUMS), Pasdaran Avenue, Birjand 9713643138, Iran; 4 Department of Clinical Toxicology and Forensic Medicine, Faculty of Medicine, Birjand University of Medical Sciences (BUMS), Ghaffari Avenue, Birjand 9717853577, Iran

## Letter to the editor

The efficacy of oral activated charcoal (AC) for the adsorption of drugs and poisons has been widely described in the literature [1]. AC can prevent systemic absorption of drugs if administrated within 1–2 h of ingestion and possibly longer after ingestion of sustained-release preparations or drugs that delay gastric emptying, such as opioids or antimuscarinic drugs. Since routine use of AC is discouraged [1], it is important to consider the risks and benefits of AC on a drug-by-drug basis. This brings us to the question of whether AC should be administrated to patients with tramadol overdose?

Balancing the hazards of tramadol poisoning *versus* the potential risks of charcoal administration is important for answering this question. In general, AC is considered to be a benign type of management, but some risks are associated with its use. Many patients vomit while some aspirate gastric contents into the lungs, causing pneumonitis [2-4]. Significant predictive factors for aspiration pneumonitis after drug overdose include a Glasgow Coma Scale score of <15, emesis, seizure, and ingestion of tricyclic antidepressants [2]. The mortality for patients with aspiration pneumonitis has been reported to be 8.5% compared with 0.4% for those without aspiration pneumonitis, with patients with aspiration pneumonia having a significantly longer hospitalization [2].

In recent years, tramadol poisoning has become one of the most common causes of admissions to emergency departments in Iran [5-11]. Important complications of tramadol poisoning include seizures as well as depression of the central nervous system (CNS) and respiratory system. It has been reported that 15% to 35% of hospital referred patients with tramadol poisoning experience seizures [5-7]. The lowest dose associated with seizures was 200 mg [5] in one study and 300 mg in another [9].

Seizures, CNS depression, and loss of protective airway reflexes are serious risk factors for pulmonary aspiration, and render the administration of AC very hazardous. Moreover, most seizures due to tramadol poisoning occur within the first 6 h of ingestion, with some studies reporting onset of seizures within the first 2 h [6]. AC is expected to be the most effective agent for preventing the systemic absorption of drugs if given within 1–2 h of ingestion.

Seizure onset may occur early after tramadol ingestion, making pulmonary aspiration of gastric contents and AC more likely. We believe that this treatment should be avoided unless the patient is already intubated with an endotracheal tube. Moreover, the risk and benefit of administration of AC should be considered in these patients to avoid potential aspiration pneumonitis unless the patient is already intubated and the airways are secured.
